# Habitual responding for alcohol depends upon both AMPA and D2 receptor signaling in the dorsolateral striatum

**DOI:** 10.3389/fnbeh.2014.00301

**Published:** 2014-09-02

**Authors:** Laura H. Corbit, Hong Nie, Patricia H. Janak

**Affiliations:** ^1^School of Psychology, The University of SydneySydney, NSW, Australia; ^2^Department of Neurology, University of California at San FranciscoSan Francisco, CA, USA; ^3^Department of Psychological and Brain Sciences, Johns Hopkins UniversityBaltimore, MD, USA

**Keywords:** habit learning, glutamate, dopamine, devaluation, dorsal striatum, rat

## Abstract

Chronic alcohol self-administration leads to alcohol-seeking behavior that is habitual and insensitive to changes in the value of the earned alcohol. Such behavior has been shown to rely on the dorsolateral region of the striatum in rats but the specific pharmacological control of output from this region is not yet understood. In the following experiments rats were trained to self-administer unsweetened 10% (v/v) ethanol in daily sessions for 8 weeks prior to testing for sensitivity to outcome devaluation. We examined the role of glutamatergic AMPA-receptor activation by testing the effects of the antagonist NBQX (0.3 and 1.0 μg/μl) infused specifically into the dorsolateral striatum (DLS) before devaluation testing. In a separate group of rats we examined the role of dopaminergic D2-receptor activation using the D2-receptor antagonist raclopride (0.2 and 1.0 μg/μl) infused into the DLS before devaluation testing. Following control (saline) infusions rats’ lever-press performance was insensitive to devaluation of ethanol thus showing evidence of habitual responding. NBQX and racolpride each restored goal-directed control of responding at doses that did not impair overall lever-press rates. These data demonstrate that expression of habitual alcohol seeking relies on glutamatergic inputs to the DLS and D2 receptors within the DLS.

## Introduction

Growing evidence from studies using both natural and drug rewards suggests that control of reward-seeking behaviors changes over the course of extended training (Adams, 1982; Zapata et al., 2010; Corbit et al., 2012). Early in training, performance is flexible and goal-directed whereas following more extended practice, performance becomes more rigid or habitual. This shift has been demonstrated to occur sooner when a drug serves as the outcome of instrumental responding or when animals have been independently exposed to drugs, including alcohol, amphetamine, and cocaine (Nelson and Killcross, 2006; Corbit et al., 2012, 2014; LeBlanc et al., 2013). In the context of drug use and exposure, this accelerated shift may reflect some of the early stages of loss of control over reward-seeking that is characteristic of substance use disorders and thus, there has been increasing interest in defining the mechanisms of striatal-based learning as well as any neural adaptations that may promote this shift in behavioral control.

The outcome devaluation task can be used to differentiate between goal-directed and habitual performance. In this task, animals are pre-fed their earned outcome, for example, alcohol to satiety, thus decreasing its value relative to a control condition where they are pre-fed a different outcome (e.g., sucrose). Performance of the response that previously earned alcohol is then tested in extinction. If responding tracks the modified value of the outcome, performance is said to be goal-directed. If responding is insensitive to changes in outcome value, this provides evidence for habitual performance.

We have recently demonstrated that following relatively limited (2 weeks) alcohol self-administration; performance is sensitive to devaluation but that following more extended training (8 weeks) performance is no longer sensitive to devaluation. Importantly, animals receiving equivalent training with sucrose reward remained sensitive to outcome devaluation, thus showing evidence of goal-directed responding, unless they were drinking alcohol in the home cage during the training period. These findings provide evidence that alcohol seeking more readily becomes habitual after extended training and/or prolonged exposure to the drug (Corbit et al., 2012).

Notably, findings, from our own and others’ work, support an important role for the dorsal striatum (DS) in drug and alcohol self-administration (Vanderschuren and Everitt, 2005; Jeanblanc et al., 2006; Wang et al., 2007; Belin and Everitt, 2008; Corbit et al., 2012) and indicate that behavioral control shifts from medial to lateral across the course of training. For example, inactivation of the dorsolateral striatum (DLS) has little effect when tests are performed following limited training when performance is goal directed. In contrast, inactivation of the DLS following extended training blocks the expression of habitual alcohol seeking restoring goal-directed control (Corbit et al., 2012). Here we sought to further explore the neuropharmacological basis for this effect.

The striatum is comprised primarily of GABAergic medium spiny projections neurons. These neurons require excitatory glutamatergic input (from cortex, thalamus, etc.) to fire action potentials (Wilson et al., 1983; Wickens et al., 2003). A recent study found that habitual performance increases activity in both the DLS and associated cortical regions (somatosensory and motor cortices), as measured by c-Fos immunohistochemistry (Furlong et al., 2014). Additionally, there are reports that activation of sensorimotor cortex increases following extended training of a motor skill (Karni et al., 1995; Floyer-Lea and Matthews, 2005). Thus the shift in control of responding from DMS to DLS across extended training likely corresponds to increased activation of at least some subsets of medium spiny neurons by glutamatergic afferents and blockade of glutamatergic input to the DLS should attenuate habitual responding for alcohol. Therefore, we examined the role of glutamatergic inputs to the DLS in the expression of habitual alcohol seeking by testing the effects of infusions of the AMPA receptor antagonist, NBQX, prior to devaluation.

Neuronal activity in the DLS is also shaped by dopaminergic inputs from the substantia nigra pars compacta. Dopamine is considered a critical contributor both to learning and expression of instrumental responding (Salamone et al., 1997; Schultz, 2007; Wickens et al., 2007) as well as a mediator of drug seeking characterized as habitual or compulsive (Volkow et al., 2006; Belin and Everitt, 2008). Direct evidence for a role of dopamine in habit learning comes from demonstrations that 6-OHDA lesions of the DA input to the DLS block the development of habitual responding in that well-trained performance remained sensitive to devaluation (Faure et al., 2005). Hence dopamine release may be a critical component in habitual alcohol-seeking behavior mediated by the DLS. Previous studies have generally tested the effects of lesions (Faure et al., 2005) or non-selective dopamine antagonists (e.g., α-flupenthixol; Dickinson et al., 2000; Belin and Everitt, 2008) and so the specific role of different classes of dopamine receptors in habit learning is not known.

Dopamine D2 receptor expression shows a gradient within the DS with these receptors being more prominent in more lateral striatal regions. In contrast, D1 receptors are expressed relatively evenly across the DS (Joyce et al., 1985; Yin et al., 2009). Evidence suggests performance of a newly-learned response is dependent on both D1- and D2-expressing neurons of the direct and indirect pathways (Choi et al., 2005; Yin et al., 2009). Following extended training, performance becomes independent of D1 receptors, while D2 activation remains important for normal performance (Yin et al., 2009). Thus, we hypothesized that habitual instrumental performance following extended training may similarly rely on D2 receptor activation. While Yin et al. (2009) examined the effects of systemic administration of dopamine antagonists, given the demonstrated role of the DLS is habitual performance, we tested the effects of intra-DLS infusions of the selective D2-receptor antagonist, raclopride, prior to devaluation testing in rats with extensive alcohol self-administration training to examine whether D2 receptors specifically within the DLS are necessary for the expression of habitual alcohol-seeking.

## Materials and methods

### Subjects and apparatus

Thirty-four naïve male Long-Evans rats (Harlan, Indianapolis, IN) weighing approximately 350 g at the beginning of the experiment were singly-housed and had free access to food and water in the home cage. All procedures were approved by the Institutional Animal Care and Use Committee of the EGCRC and conform to the standards stipulated by the National Institutes of Health Office of Laboratory Animal Welfare. Training and testing took place in Med Associates (East Fairfield, VT) operant chambers described previously (Corbit et al., 2012).

### Alcohol acclimation in the home cage

Because of the aversive taste of alcohol rats are initially relatively reluctant to consume unsweetened alcohol voluntarily. To familiarize them with alcohol, rats initially were given free access to 10% ethanol (10E; v/v) in filtered water in the home cage, for 24 h a day for 14 days, followed by 14 days of 1 h access to 10E at the time that training would subsequently occur. Water was always available in a separate bottle fixed to the home cage. Rats were weighed daily and consumption recorded.

### Instrumental training

Rats underwent a single 30 min magazine training session wherein 10E was delivered under a random time-60 s schedule. Rats were next trained to make a lever-press response to deliver small aliquots (0.1 ml) of 10E in 60 min sessions. The first 2 days of training were under a continuous reinforcement schedule; reinforcement was then shifted to a random ratio (RR) two schedule for 3 days, followed by a RR3 schedule. Animals failing to respond at levels sufficient to achieve alcohol intake of at least 0.3 g/kg for 5 out of 7 days a week were excluded from the study (9 animals excluded according to this criterion leaving 25 animals which were subsequently assigned to either the NBQX (*N* = 13) or raclopride (*N* = 12) experiment). The reward receptacle was examined at the end of each session to ensure that the earned rewards were consumed; after the initial 3 training days this was always the case.

### Surgery

Surgery was performed after approximately 7 weeks of training. Stereotaxic surgery was conducted under isoflorane anesthesia to implant 26 gauge guide cannulae (Plastics One, Roanoke, VA) targeting the DLS (AP: +1.2 mm, ML: +/−3.4 mm, DV: –1.0 mm; coordinates relative to bregma). Guide cannulae tips were positioned 3 mm dorsal to the intended infusion site; thus, final DV coordinates for the infuser tips was −4.0 ventral to dura. Animals were given a week to recover from surgery and resumed training for 1 week prior to devaluation testing.

### Devaluation testing

For each test, rats were divided into two groups, devalued and non-devalued. For the devalued condition, rats were given 45-min of free access to 10E in the home cage. These parameters typically result in average consumption of 5 ml of 10E and corresponding alcohol levels of 0.85 g/kgs (Corbit et al., 2012). For the non-devalued condition, rats were given 45-min free access to 1% sucrose (wt/vol; 1S; this concentration was chosen as it produces consumption volumes similar to those found with 10E; Corbit et al., 2012). A consumption criterion of 3 ml was required for an animal’s data to be included. Immediately following home-cage pre-feeding, rats received an infusion (as described below) and were tested for lever-press responding in a 10-min extinction test. Following this first test, rats received 2 days of retraining and were tested again such that rats that had received the devaluation treatment now received the non-devalued treatment and vice versa. Additional pairs of tests were completed in the same fashion to allow testing of each rat under each dose.

### The role of DLS AMPA receptors in habitual alcohol seeking

Each animal underwent a total of six tests to allow testing in both the devalued and non-devalued conditions under saline and two doses of the AMPA-receptor antagonist (order counterbalanced for dose and devaluation condition). The AMPA antagonist used was NBQX (Sigma, St Louis, MO) and 0.3 μl per hemisphere of two doses (0.3 and 1.0 μg/μl) were delivered via infusion cannulae (33 gauge; Plastics One) extending 3 mm below the guide cannula tip at a rate of 0.3 μl/min 10 min prior to test. The infusion cannulae were left in place for at least 1 min after the completion of the infusion to allow for diffusion of the drug away from the tip.

### Consumption tests of the efficacy of the devaluation treatment

It is possible that after 8 weeks of self-administration training the satiety treatment may lose its efficacy due to development of tolerance or other factors. To address this concern, on separate days, rats were given 45-min of free access to either 10E or 1% sucrose in the home cage. These bottles were removed and 10 min later (approximately the time the extinction test would have occurred following infusions) animals were presented with a fresh bottle of 10E. Consumption was recorded. We predicted that if the devaluation treatment was effective, animals would consume less alcohol following previous consumption of alcohol (devalued condition) compared to previous consumption of sucrose (non-devalued condition).

### Effects of NBQX on consumption

To address whether NBQX might have any non-specific effects on alcohol consumption we examined the effects of NBQX treatment on homecage drinking. Rats received an infusion of 0.3 μg/μl NBQX and were returned to their homecage and a bottle of alcohol was provided for 1 h. Bottles were weighed at 30 and 60 min.

### The role of DLS D2 receptors in the expression of habitual alcohol seeking

Separate animals underwent acclimation training and surgery as described above. Each animal underwent a total of six tests to allow testing in both the devalued and non-devalued conditions under saline and two doses of the D2 receptor antagonist raclopride (0.2 and 1.0 μg/μl; order of devaluation condition and dose was counterbalanced). Saline or two doses of raclopride (0.3 μl; Sigma, St Louis, MO) were delivered via infusion cannulae 10 min to devaluation testing as described above.

### Histology

Coronal sections (50 μm) of formalin-fixed tissue were sliced, mounted, and stained with Nissl stain, to allow verification of cannulae placement.

### Data analysis

Data were analyzed in repeated measures analysis of variance (ANOVA) as appropriate with the within-subjects factors of devaluation (devalued vs. non-devalued) and drug dose.

## Results

### The role of DLS AMPA receptors in habitual alcohol seeking

#### Histology

**Figure 1:**
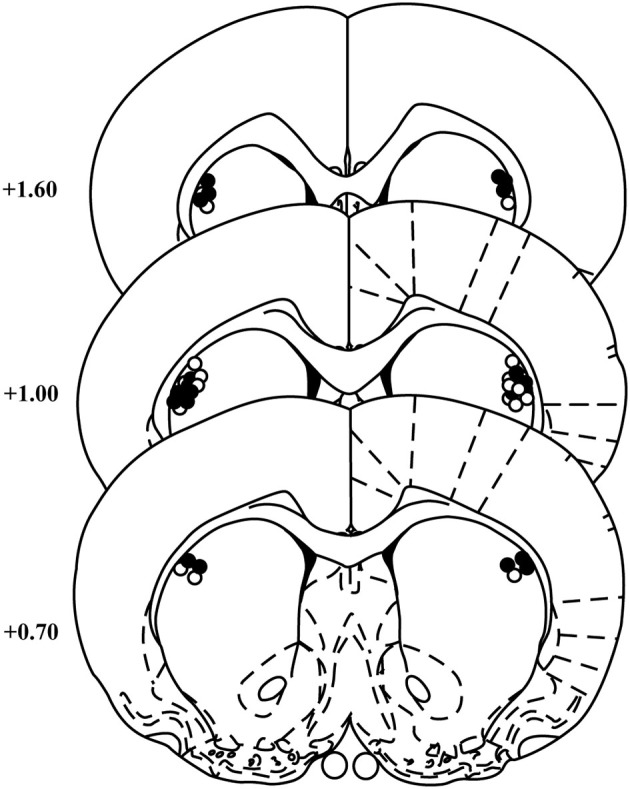
**Cannula placements within the DLS**. Schematic representation of cannulae placements in the DLS for animals included in the NBQX (open circles) and raclopride (black circles) experiments (templates adapted from Paxinos and Watson, 1998). Numbers indicate the distance from bregma in anterior–posterior plane. *N* = 11 and 12 for NBQX and raclopride experiments, respectively.

Cannula placements for animals included in the behavioral analyses for the two experiments were similar and are summarized in Figure 1. Two rats were excluded from the NBQX experiment for misplaced cannulae leaving 11 rats in the final analyses.

#### Training

**Figure 2:**
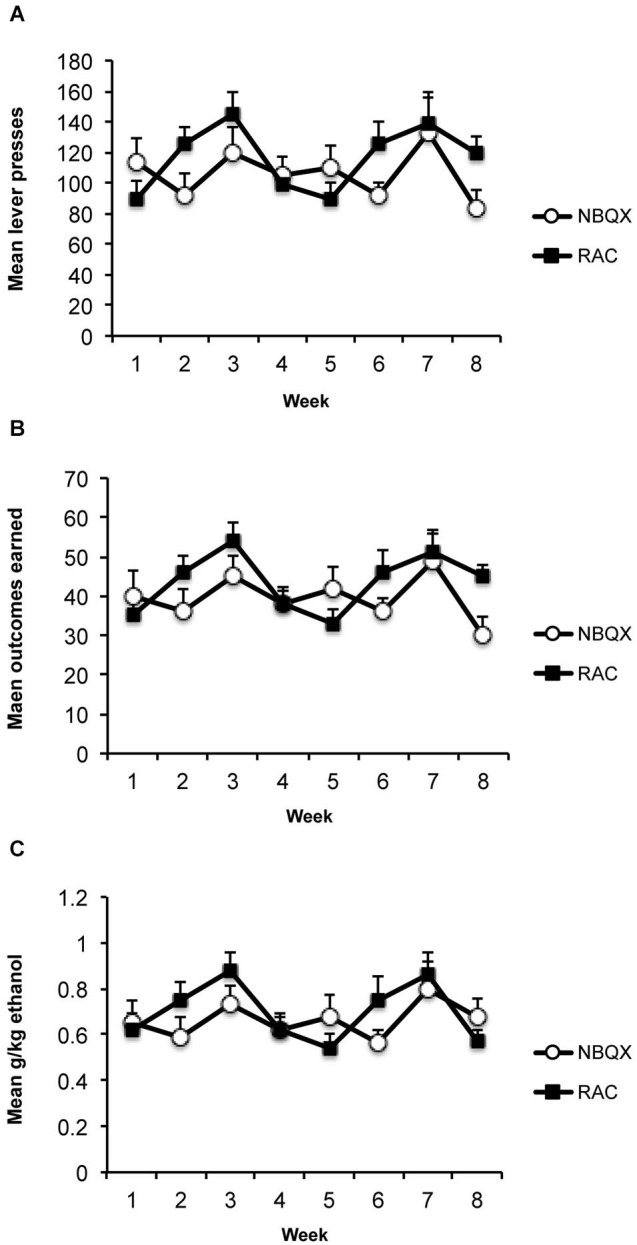
**Training data**. **(A)** Mean (+/−SEM) lever presses, **(B)** outcomes earned, and **(C)** g/kg alcohol across weeks of instrumental training prior to devaluation testing.

The average consumption during the 1 h access in the home cages was 2.65 ml (+/−SEM; 0.35 ml) which produced an average alcohol level of 0.41 g/kg (+/−0.055). Average responding, outcomes earned and g/kg alcohol levels across the 8 weeks of training are presented in Figure 2. Averaging across the last 3 training days before the first test the rats made 121 (+/−12.1) active lever presses, 2.1 (+/−0.7) inactive lever presses, earned 45 (+/−4.3) outcomes producing an average alcohol level of 0.7 (+/−0.07) g/kg.

#### Devaluation testing

**Figure 3:**
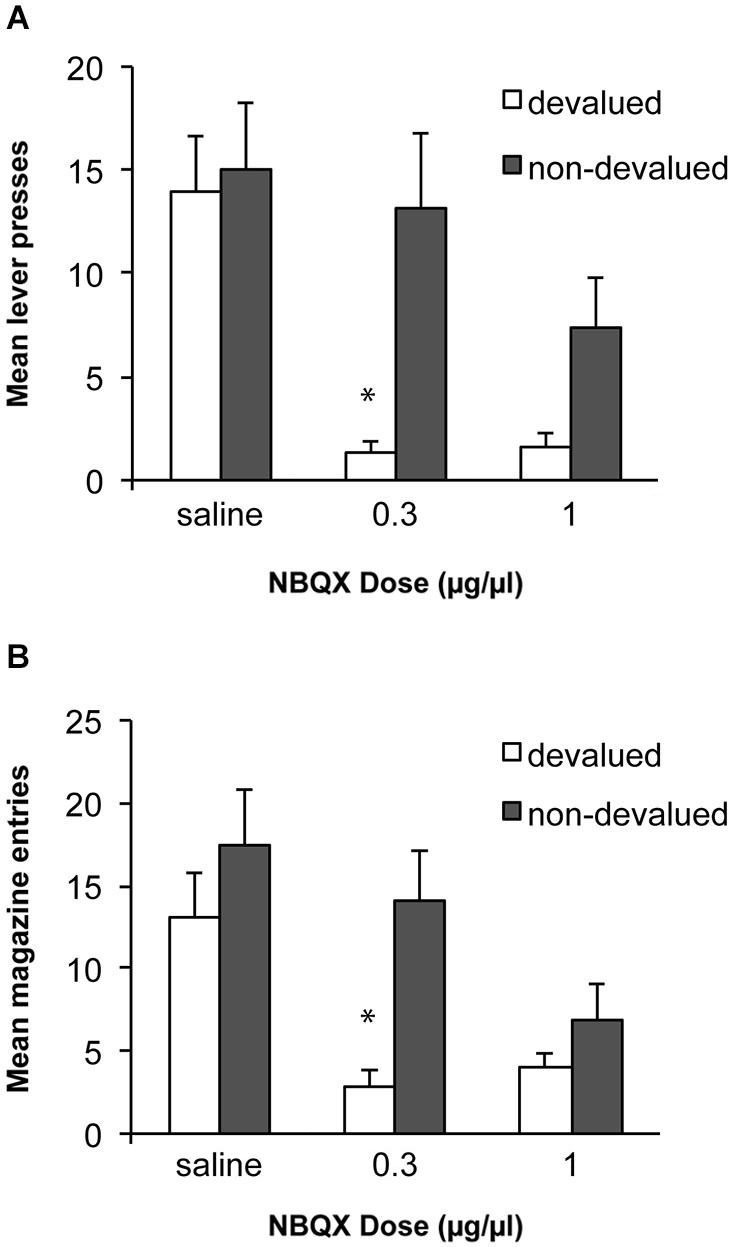
**Effects of NBQX infusion into the DLS on sensitivity to outcome devaluation**. **(A)** Following 8 weeks of ethanol self-administration lever-press responding was insensitive to devaluation following saline infusions. Infusion of 0.3 μg/μl NBQX restored sensitivity to devaluation. Infusion of the higher dose (1.0 μg/μl) produced a marginal devaluation effect but overall responding was also lower. * indicates *p* < 0.05. **(B)** Mean magazine entries during the devaluation tests which show a similar pattern to lever-press performance.

Based on previous work with this paradigm we predicted that alcohol self-administration after 8 weeks of training would be habitual and insensitive to devaluation of the alcohol outcome. We tested whether antagonism of AMPA receptors within the DLS, thus blocking fast excitatory transmission to this region, would block the expression of the acquired habit and restore goal directed performance. Data from these tests are presented in Figure 3A. An ANOVA examining sensitivity to devaluation after infusions of saline or NBQX demonstrated a significant effect of devaluation [*F*_(1, 10)_ = 5.4, *p* < 0.05], of drug [*F*_(2, 20)_ = 9.2, *p* < 0.01] and an interaction between these factors [*F*_(2,20)_ = 4.5, *p* < 0.05]. Simple effects analyses revealed that following an infusion of saline rats were not sensitive to devaluation of alcohol and responded similarly under the devalued and non-devalued conditions [*F*_(1,10)_ = 0.3, *p* > 0.05]. In contrast, infusion of 0.3 μg/μl of NBQX restored sensitivity to devaluation, such that rats decreased responding following devaluation of alcohol compared to the non-devalued condition [*F*_(1,10)_ = 9.0, *p* < 0.05]. Infusion of a higher dose of NBQX (1.0 μg/μl) produced a marginal devaluation effect [*F*_(1,10)_ = 4.6, *p* = 0.057] but this dose also decreased responding overall which may have contributed to this effect. To examine a potential general effect on motor behavior following infusions particularly in the devalued condition where there is potential for an interaction with the pharmacological effects of the alcohol consumed, we examined magazine entries during the tests. These data are presented in Figure 3B. Overall, the magazine response showed the same pattern as the lever-press response with a significant effect of devaluation [*F*_(1, 10)_ = 6.2, *p* < 0.05], of drug [*F*_(2, 20)_ = 10.2, *p* < 0.01] and an interaction between these factors [*F*_(2,20)_ = 3.8, *p* < 0.05]. Sensitivity to devaluation was only observed following the low dose of NBQX [*F*_(1,10)_ = 12.6, *p* < 0.01]. Following the high dose of NBQX responding did not differ significantly between devalued and non-devalued conditions [*F*_(1,10)_ = 1.7, *p* > 0.05] and responding in the devalued condition was numerically higher than responding following devaluation and the lower dose of NBQX which would not be anticipated if NBQX was producing a deficit because of an interaction with the pharmacological effects of alcohol consumed in the devaluation tests.

**Figure 4:**
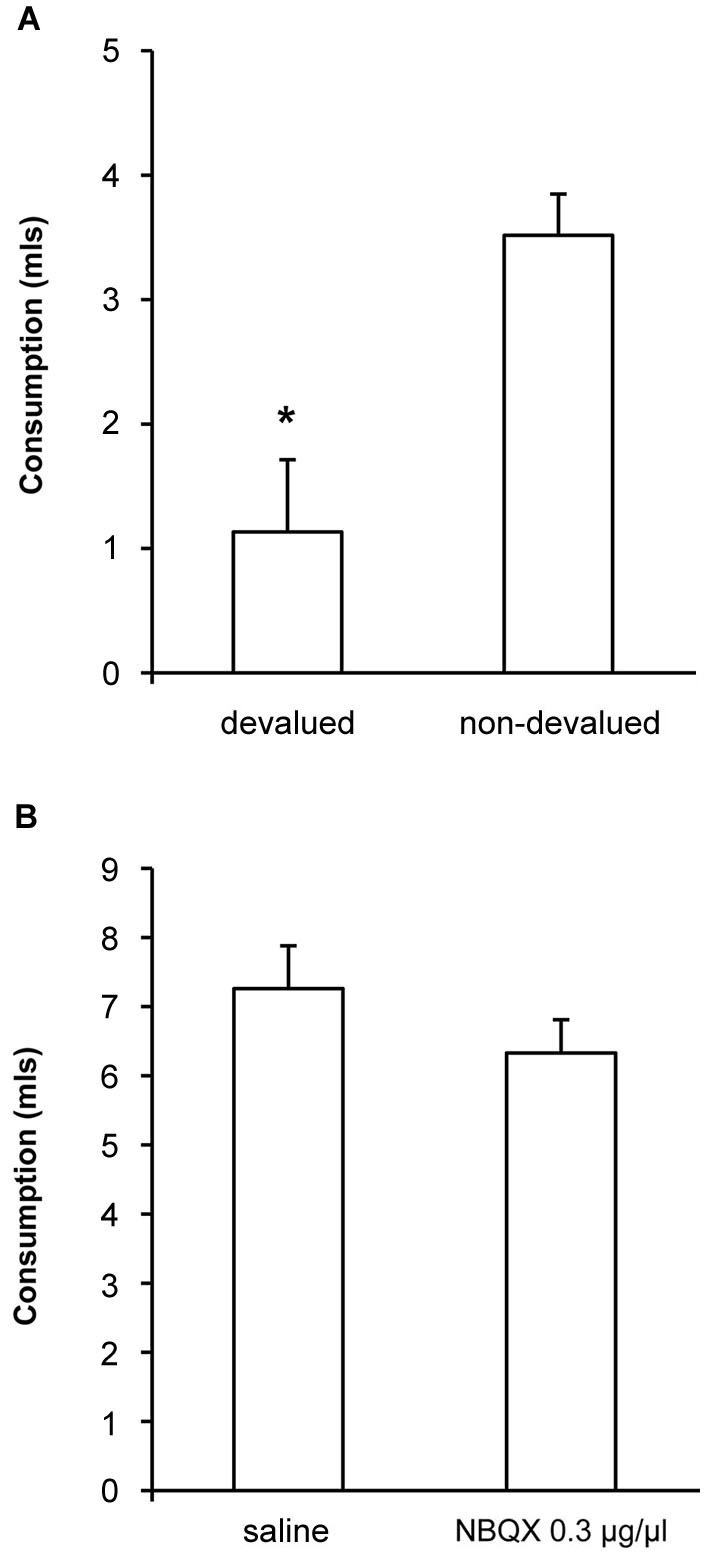
**Effects of NBQX on sensitivity of consumption to outcome devaluation and on home cage drinking**. **(A)** When consumption of ethanol following pre-feeding of ethanol (devalued) or sucrose (non-devalued) was measured, a significant devaluation was detected demonstrating that the specific satiety treatment itself was effective but that this change in outcome value was not translated into lever-press performance under control conditions (see saline condition in Figure 3). **(B)** Ethanol consumption in the home cage following infusion of either saline or 0.3 μg/μl NBQX was equivalent indicating that NBQX treatment itself did not somehow change the rats’ willingness to consume ethanol. * indicates *p* < 0.05.

To confirm that the devaluation treatment itself was effective following extended alcohol exposure we measured consumption of ethanol following pre-feeding of ethanol (devalued) or sucrose (non-devalued). As shown in Figure 4A, we found a significant devaluation [*F*_(1, 10)_ = 8.4, *p* < 0.05] demonstrating that the specific satiety treatment itself was effective, but that this change in outcome value did not direct lever-press performance under control conditions. We also examined whether our most effective dose of NBQX might have any non-specific effects on alcohol consumption by examining the effects of NBQX infusion prior to 1 h access to alcohol in the home cage. Consumption following infusion of either saline or 0.3 μg/μl NBQX is shown in Figure 4B and was equivalent [*F*_(1,10)_ = 1.9, *p* > 0.05] indicating that NBQX treatment itself did not somehow make alcohol aversive or alter satiety or willingness to consume alcohol. Together, these findings suggest that the restoration of sensitivity to devaluation following NBQX was not secondary to altered satiety, intoxication or non-specific motor effects.

### The role of DLS D2 receptors in the expression of habitual alcohol seeking

#### Histology

All cannulae were placed within the DLS (*N* = 12).

#### Training

The average consumption during the 1 h access in the home cages was 3.1 ml (+/−SEM; 0.48 ml) which produced an average alcohol level of 0.50 g/kg (+/−0.08). Average responding, outcomes earned and g/kg alcohol levels across the 8 weeks of training are presented in Figure 2. Averaging across the last 3 training days before the first test the rats made 101 (+/−12.3) active lever presses, 1.4 (+/−1.1) inactive lever presses, earned 37 (+/−43.9) outcomes producing an average alcohol level of 0.58 (+/−0.06) g/kg.

#### Devaluation testing

**Figure 5:**
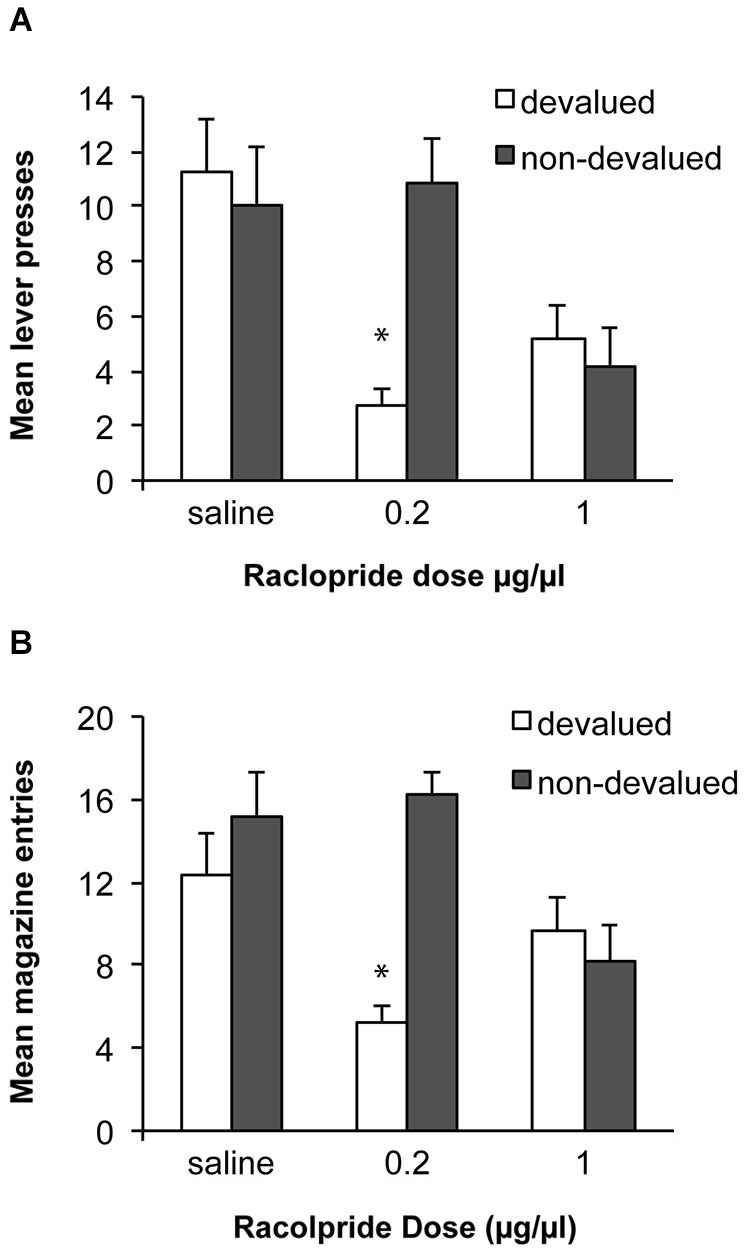
**Effects of raclopride infusion into the DLS on sensitivity to outcome devaluation**. **(A)** Following 8 weeks of ethanol self-administration lever-press responding was insensitive to devaluation following saline infusions. Infusion of the 0.2 μg/μl dose of raclopride restored sensitivity to devaluation. After infusion of the higher dose (1.0 μg/μl) sensitivity to devaluation was again lost. * indicates *p* < 0.05. **(B)** Mean magazine entries during the devaluation tests.

Based on the previous demonstration that performance of a well-learned skill relies on D2 receptor activity in the DLS (Yin et al., 2009), we predicted that alcohol self-administration after 8 weeks of training would be habitual and that antagonism of D2 receptors within the DLS, would block the expression of the acquired habit and restore goal-directed performance. Data from the devaluation tests are shown in Figure 5A. An ANOVA examining sensitivity to devaluation after infusions of saline or raclopride demonstrated that overall there was no effect of devaluation [*F*_(1, 11)_ = 1.9, *p* > 0.05], but that there was a significant effect of drug [*F*_(2, 22)_ = 5.2, *p* < 0.05] and an interaction between these factors [*F*_(2,22)_ = 11.9, *p* < 0.01]. Simple effects analyses revealed that following an infusion of saline rats were not sensitive to devaluation of alcohol and responded similarly under the devalued and non-devalued conditions [*F*_(1,11)_ = 0.3, *p* > 0.05]. In contrast, infusion of 0.2 μg/μl of raclopride restored sensitivity to devaluation and rats decreased responding following devaluation of alcohol compared to the non-devalued condition [*F*_(1,11)_ = 23.8, *p* < 0.01]. Infusion of a higher dose of raclopride (1.0 μg/μl) did not produce a significant devaluation effect [*F*_(1,11)_ = 0.3, *p* > 0.05] but this dose also decreased responding in general. As above, to examine a potential motor effect particularly in the devalued condition where there is potential for an interaction between raclopride and the pharmacological effects of the alcohol consumed, we examined magazine entries during the tests (see Figure 5B). There was an effect of devaluation [*F*_(1, 11)_ = 10.3, *p* < 0.05], a significant effect of drug [*F*_(2, 22)_ = 3.5, *p* < 0.05] and an interaction between these factors [*F*_(2,22)_ = 9.7, *p* < 0.01]. Simple effects analyses indicated a significant effect of devaluation only after the low dose of raclopride [*F*_(1,11)_ = 100.8, *p* < 0.01]. Following the high dose of raclopride, responding did not differ significantly between devalued and non-devalued conditions [*F*_(1,11)_ = 0.4, *p* > 0.05] and responding in the devalued condition was numerically higher than responding following devaluation and the lower dose which would not be anticipated if raclopride was producing a deficit because of an interaction with the pharmacological effects of alcohol consumed in the devaluation tests.

It is unclear whether the effects of the higher dose are simply due to greater inhibition of D2 receptors or additional recruitment of different classes of D2 receptors. That is, in addition to affecting postsynaptic D2 receptors on medium spiny neurons of the indirect pathway, raclopride could also inhibit autoreceptors and interfere with feedback mechanisms and dopamine levels (Anzalone et al., 2012). The role of these different classes of D2 receptors in habit learning and expression will be an interesting area for future study.

## Discussion

The data presented here provide further evidence that with extended training, performance of an alcohol-seeking response becomes habitual and no longer tracks the current value of the outcome it produces. Further, we demonstrate that this habitual performance relies on both AMPA and D2 receptors within the DLS as treatment with either the AMPA-receptor antagonist NBQX or the selective D2-receptor antagonist raclopride suppressed expression of habitual performance thus restoring goal-directed control.

The observation of habitual responding for alcohol can not easily be accounted for by insensitivity to the devaluation manipulation after prolonged alcohol exposure, as rats selectively decrease consumption of alcohol following a previous opportunity to consume alcohol, compared to following consumption of sucrose. Thus, factors such as tolerance which may change across the course of extended alcohol exposure do not readily explain the insensitivity of lever-press performance to devaluation. Rather, it appears that animals no longer flexibly utilize the current value of the earned alcohol to control alcohol-seeking behaviors.

The effects of NBQX confirm that glutamatergic inputs to the DLS are important for driving habitual performance. This is consistent with a recent report that habitual performance increases activity in both the DLS and associated cortical regions (somatosensory and motor cortices) measured by c-Fos immunohistochemistry (Furlong et al., 2014) and reports that activation of sensorimotor cortex increases following extended training of a motor skill (Karni et al., 1995; Floyer-Lea and Matthews, 2005). Thus glutamatergic inputs from cortex likely contribute to habitual performance and these were blocked by NBQX. Similarly, the effects of raclopride suggest that dopaminergic modulation of DLS activity is also important for the expression of habitual responding. One caveat to this conclusion is that it is possible that the raclopride treatment interacted with the pharmacological effects of alcohol in the devaluation tests to exacerbate any intoxicating or motor effects of alcohol. While we did not assess the effects of raclopride on alcohol consumption in the current study, previous work has shown that raclopride administered to the nucleus accumbens core has little effect on ethanol consumption at doses that do impair an alcohol-seeking response (Czachowski et al., 2001) and since the tests of habitual performance were conducted in extinction, without alcohol present, we believe any effects of raclopride related to consumption are unlikely to account for the observed effects. Indeed, magazine approach increased in the devalued condition after the higher dose of raclopride and thus the restoration of a devaluation effect is difficult to explain in terms of non-specific effects of the drug on intoxication or motor performance.

The ability of the D2-receptor antagonist raclopride to block habitual responding is however consistent with the findings of Yin et al. (2009) who demonstrated that systemic administration of a D2 antagonist disrupted performance of a motor skill after extended training. Yin et al. (2009) found that antagonism of D1 receptors during the initial stages of training disrupted rotorod performance but the same treatment was without effect after extended training. This finding is in agreement with previous demonstrations that D1 receptor activation is important for initial learning but plays a decreasing role as training progresses. For example, Choi et al. (2005) found that treatment with a D1 antagonist disrupted both cued and non-cued approach to a food receptacle after limited (3 days) training but was without effect on cued responding following more extended training (16 days) although effects on spontaneous responses remained. This is consistent with the suggestion that compromised dopamine transmission does not produce pure motor deficits but rather impairs the ability to generate voluntary motor acts in the absence of external eliciting stimuli (Jahanshahi and Frith, 1998; Choi et al., 2005). While there was no direct test of whether performance was in fact habitual in the study by Choi and colleagues (i.e., insensitive to outcome devaluation or changes to the response-outcome contingency) the selective effect on cued responding could be explained by a S-R (stimulus-response) learning mechanism like that argued to underlie habitual performance and which the authors suggest may become dopamine-independent over the course of extended training.

Stimuli, and particularly the context in which rats were trained and tested may contribute importantly to the observed effects. There is direct evidence that alcohol-paired environmental stimuli can promote habitual behavior. For example, Ostlund et al. (2010) found that when rats were tested in a saline-paired context they showed a significant devaluation effect. However, when the same rats with the same training history were tested in a context that had been paired with alcohol they were insensitive to outcome devaluation indicating habitual responding and demonstrating that alcohol-predictive cues can disrupt decision making. Similar results have bee reported with more discrete alcohol cues in humans (Hogarth et al., 2013). Discrete cues can also trigger and maintain responding for alcohol and other rewards (Lê and Shaham, 2002; Loeber et al., 2006; Corbit and Janak, 2007a) and many of these stimulus effects rely on the DLS (Vanderschuren et al., 2005; Volkow et al., 2006; Corbit and Janak, 2007b). Thus it is possible that the effects of either NBQX or raclopride relate to an ability to suppress the influence of environmental stimuli, such as the training context, which over the course of training had become paired with alcohol, and thus a remove context-mediated bias towards habitual performance.

It has been suggested that over the course of extended training control of responding is less dependent on dopamine because it shifts to brain regions outside the striatum. For example, single-unit activity in the sensorimotor striatum has been reported to decrease over the course of extended training (Carelli et al., 1997) as does phasic dopamine release in this region (Willuhn et al., 2014). This finding is difficult to reconcile with multiple demonstrations that lesions or inactivation of the DLS abolish habitual performance indicating that the DLS is necessary for the acquisition and expression of response habits (Yin et al., 2004; Corbit et al., 2012). Others have reported decreases in the number of sensorimotor striatal neurons showing task-relevant phasic activity over the course of training, but also that a small group of neurons in this region continue to show task-relevant activity and that the magnitude of neural responses grows with extended training (Barnes et al., 2005; Tang et al., 2007). These demonstrations may reflect weakening of non-task-relevant corticostriatal inputs (through LTD) and/or lateral inhibition of competing striatal neurons by the neurons that have been potentiated over the course of learning (Ashby et al., 2010).

In contrast to the diminishing role of D1 receptor activity following extended training (Choi et al., 2005), several studies have demonstrated that a D1 antagonist (SCH23390) given either during instrumental training (Nelson and Killcross, 2013) or prior to devaluation testing (Furlong et al., 2014) can suppress habitual performance. Notably, in those studies repeated amphetamine or chronic access to a palatable food was given to animals prior to instrumental training and subsequently was found to produce more rapid habit learning than seen in controls. It is not currently known whether the rapid habit learning that follows certain previous experiences including exposure to potent rewards such as drugs rely on the same mechanisms as habit learning following extended training and the role of specific dopamine receptors may differ in these situations. Indeed, in the present study, there are two factors that may contribute to habitual control of performance and consideration of each is important for understanding the role of dopamine in behavioral control; first, rats had chronic exposure to alcohol, and second, they also underwent extensive training. Extended training itself can promote habitual control (Adams, 1982), and performance that is independent of D1 receptor activation (Choi et al., 2005; Yin et al., 2009), and as indicated by the results of Yin et al. this type of learning may recruit D2-containing neurons in the DLS. As noted above, there are now several demonstrations that mere exposure to drugs (Nelson and Killcross, 2006; Nordquist et al., 2007; Corbit et al., 2012, 2014) and other salient events (Dias-Ferreira et al., 2009; Furlong et al., 2014) can lead to subsequent learning falling rapidly under habitual control. Downregulation of D2 receptors and changes in the relative contribution of D1 vs. D2-containing neurons following long-term exposure to drugs or potent rewards (Volkow et al., 1993; Johnson and Kenny, 2010; Park et al., 2013) has been repeatedly demonstrated and may contribute to why D1 receptor antagonists reduce the expression of habitual behaviors following such treatments. Extended training itself may not produce the same adaptations in receptor expression as alcohol exposure and thus, it is possible that dopamine and its receptors will have somewhat different roles in habits produced by drug exposure and extended training. Future studies examining the role of D1 and D2 antagonists in each of these paradigms will be needed to fully understand the mechanisms underlying the shift to habitual control.

In summary, we demonstrate that extended alcohol self-administration leads to alcohol-seeking that is habitual and not sensitive to change in the value of alcohol. Sensitivity is restored following AMPA or D2 receptor antagonism in the DLS. Understanding the cognitive and neural control of alcohol-seeking particularly after extensive experience with the drug and behaviors involved in procuring it will be important for understanding the loss of control that is a hallmark of substance use disorders including alcoholism.

## Conflict of interest statement

The authors declare that the research was conducted in the absence of any commercial or financial relationships that could be construed as a potential conflict of interest.
